# Dynamic Interaction Between SARS-CoV-2 and Influenza A Virus Infection in Human Respiratory Tissues and Cells

**DOI:** 10.3390/microorganisms13050988

**Published:** 2025-04-25

**Authors:** John C. W. Ho, Kachun Ng, Rachel H. H. Ching, Malik Peiris, John M. Nicholls, Michael C. W. Chan, Kenrie P. Y. Hui

**Affiliations:** 1 School of Public Health, Li Ka Shing Faculty of Medicine, The University of Hong Kong, Pokfulam, Hong Kong SAR, China; cwjohn@connect.hku.hk (J.C.W.H.); hhching@hku.hk (R.H.H.C.); malik@hku.hk (M.P.); mchan@hku.hk (M.C.W.C.); 2 Centre for Immunology and Infection (C2i), Hong Kong Science Park, Shatin, Hong Kong SAR, China; 3 Department of Pathology, School of Biomedical Sciences, Li Ka Shing Faculty of Medicine, The University of Hong Kong, Pokfulam, Hong Kong SAR, China; nicholls@pathology.hku.hk

**Keywords:** influenza, SARS-CoV-2, co-infection, innate immune response, respiratory explant

## Abstract

With the concurrent circulations of SARS-CoV-2 omicron and influenza A viruses in the community, there is evidence showing co-infection with both viruses. However, disease severity may vary due to the complex immunity landscape of the patients and the neutralizing antibody waning status. The intrinsic dynamic relationship and pathological significance for such co-infections remain largely unknown. The replication kinetics and innate immune responses from the co-infections of SARS-CoV-2 (Omicron BA.1 and D614G variant) and influenza A viruses (pandemic H1N1, seasonal H3N2 and highly pathogenic avian H5N1) were characterized in human respiratory tissue explants, human airway, and alveolar epithelial cells. SARS-CoV-2 reduced the replication of influenza A viruses, but not vice versa, during co-infections in human bronchial tissues and airway epithelial cells. In lung tissues, the co-infections showed minimal effects on each other, but the viral replications of the two viruses were mutually reduced except for H1N1pdm in the alveolar epithelial cells irrespective of the enhancement of the ACE2 receptor. Notably, the co-infections showed a significant upregulation of the innate immune responses of SARS-CoV-2 in comparison to single infections in both respiratory epithelial cells, suggesting that co-infections of influenza A viruses potentially lead to more severe damage to the host than SARS-CoV-2 single infections.

## 1. Introduction

Since the emergence of the Omicron lineages of severe acute respiratory syndrome coronavirus 2 (SARS-CoV-2) and the developed population immunity, the coronavirus disease 2019 (COVID-19) has exhibited signs of milder disease dissemination and clinical outcomes [1]. Nevertheless, the ongoing global pandemic has already caused a cumulative total of over 776 million reported cases, with 7 million deaths as of December 2024 [2]. Fatality is particularly severe in older adults and patients with underlying diseases such as diabetes, hypertension, and cardiovascular disease. A number of pro-inflammatory cytokines and chemokines are significantly elevated in patients with severe COVID-19, and sepsis is the most frequently observed complication followed by respiratory failure and acute respiratory distress syndrome (ARDS) [3]. Therefore, the dysregulated cytokines might play an important role in the immunopathology of COVID-19.

Upon the COVID-19 pandemic, some cases were reported to be co-infected with seasonal influenza A viruses [4] and even the highly pathogenic avian H5N1 [5]. Seasonal influenza is a common respiratory tract infection worldwide, where the major circulating influenza A viruses in humans are subtype A (H1N1) and A (H3N2). Although seasonal influenza A virus can cause severe illness or death in high-risk groups such as the elderly (≥65), young infants (≤6), and individuals with chronic medical conditions, most patients recover within a week without requiring medical attention and stay in the community during the course of infection instead of being quarantined. In our previous publication, we found that infection with the influenza A virus in human alveolar epithelial cells up-regulated the expression of angiotensin-converting enzyme 2 (ACE2) [6], which is the receptor for SARS-CoV-2 binding and host entry. ACE2 has been found to be expressed in the human upper and lower respiratory tracts and enterocytes of the small intestine and colon [7]. The presence of ACE2 in human tissues is an important factor associated with the cellular or tissue tropism of SARS-CoV-2. This association has been demonstrated by the robust infection and replication of SARS-CoV-2 in the human respiratory tract and gut enterocytes in the form of enteroids [8].

Most research regarding the co-infections of SARS-CoV-2 and influenza A viruses has focused on the early strains of SARS-CoV-2, including the wild-type and Delta, or been conducted in animal models [9,10]. More recent studies on the Omicron variant, however, have reported diverse results for its effects on interferon response and influenza A virus susceptibility in different in vitro human respiratory models. Bojkova’s report demonstrated an Omicron-induced interferon response in air–liquid interface cultures of primary human bronchial epithelial cells that suppressed the influenza virus infection [11], whilst Kim’s study reported a modest increase in influenza virus loads by Omicron co-infections in human pluripotent stem cell-induced lung organoids [12]. Therefore, it is critical to investigate the co-infection dynamics between the Omicron variant of SARS-CoV-2 and different influenza A viruses, including the HPAI, by using human ex vivo respiratory models to better mimic the existing global situation. Furthermore, it would be prudent to conduct a risk assessment on the potential co-infection of a high cytokine-inducing SARS-CoV-2 variant (D614G) with influenza A viruses. Therefore, we studied SARS-CoV-2 and influenza co-infections using relevant models and assessed the risks posed by co-infections with a high cytokine inducing SARS-CoV-2 variant. This information is crucial for preparing and responding to potential future outbreaks.

Here, we used physiologically relevant models of ex-vivo explant cultures of the human respiratory tissues, human airway, and alveolar epithelial cells to evaluate the interaction of viral replication between SARS-CoV-2 variants and influenza A viruses including seasonal, pandemic, and highly pathogenic avian influenza viruses. The innate immune responses to the co-infection and single infection were evaluated in both airway and alveolar epithelial cells.

## 2. Materials and Methods

### 2.1. Viruses

Two SARS-CoV-2 variants, including Omicron BA.1 [13] and a strong cytokine inducing strain from GISAID clade GR that carries a Spike D614G mutation as previously reported [14], were used. For the influenza A viruses, one fatal human H5N1 (A/Hong Kong/483/97), one human pandemic H1N1 (A/Hong Kong/415742/2009, H1N1pdm), and two seasonal human H3N2 (A/OK/483/08 and A/HK/4550/2016) strains were studied. SARS-CoV-2 strains were propagated and titrated using Vero E6 cells with the overexpressed TMPRSS2 gene (Vero E6/TMPRSS2), whilst the influenza A viruses were propagated and titrated with Madin–Darby canine kidney (MDCK) cells. All live virus experiments were performed in a biosafety level 3 facility at Li Ka Shing Faculty of Medicine, the University of Hong Kong.

### 2.2. Cell Culture

Vero E6/TMPRSS2 cells were provided by M. Takeda [15] and cultured in DMEM (Gibco, Frederick, MD, USA) supplemented with 10% fetal bovine serum (FBS; Gibco), 100 units/mL penicillin, and 100 µg/mL streptomycin (Gibco). MDCK cells were cultured in minimal essential medium (Gibco) supplemented with 10% FBS. Two human lung-derived epithelial cell lines, Calu-3 and the ACE2 gene-overexpressed A549 cells (A549/ACE2), were cultured in DMEM supplemented with 10% FBS. All cell cultures were maintained in 37 °C, 5% CO_2_ humidified incubators.

### 2.3. Ex Vivo Infection of Human Respiratory Explants

Human non-malignant bronchus and lung tissues were obtained from consented patients who underwent elective surgery at the Hong Kong Queen Mary Hospital (IRB approval no: UW20-862). Tissues were removed as part of the clinical care but surplus for routine diagnostic requirements as described previously [6]. Ex vivo infection of human bronchus and lung tissues were performed as described previously [6]. Briefly, bronchial and lung tissues were fragmented into small pieces and infected with influenza A and/or SARS-CoV-2 viruses by incubating with 1 mL of each virus at a titer of 1 × 10^6^ plaque forming unit/mL at 37 °C for 1 h [6,16]. The infected tissue explants were washed three times with phosphate buffered saline (PBS; Gibco), then cultured in F-12K medium (Gibco) supplemented with L-glutamine and antibiotics, and incubated in 5% CO_2_ at 37 °C for 72 h. The bronchial tissues were placed on sterile pathology sponge pads to establish an air–liquid interface condition. Culture supernatants from the infected tissue explants were collected at 1, 24, 48, and 72 h post-infection (hpi) to assess the viral replication. At 72 hpi, the infected tissue explants were harvested and were either fixed in 10% formalin for later immunohistochemistry analysis or lysed in RLT buffer (Qiagen, Hilden, Germany) for gene expression analysis.

### 2.4. Infection of Human Airway and Alveolar Epithelial Cells

Human airway epithelial cells Calu-3 were seeded onto the apical chamber of 0.4 µm pore Transwell inserts (Corning Inc., Corning, NY, USA) and cultured in an air–liquid interface condition until reaching full confluency. The Calu-3 cells were infected with influenza A and/or SARS-CoV-2 viruses at the multiplicity of infection (MOI) of 0.1 at 37 °C for 1 h, then washed with PBS and cultured in the air–liquid interface condition for 72 h. At 1, 24, 48, and 72 hpi, growth medium was added to the apical side of the Calu-3 Transwell cultures and collected for viral load determination. At 72 hpi, culture medium from both the apical and basal Transwell chambers was harvested to assess the cytokine and chemokine concentrations, and the cells were lysed with RLT buffer for gene expression analysis. Human alveolar epithelial cells A549/ACE2 were seeded onto 24-well plates and infected with influenza A and/or SARS-CoV-2 viruses at the MOI of 0.1 or 1, respectively. For sequential infection, cells were first infected with H5N1 and then SARS-CoV-2, or vice versa, at the same MOI as the above with 24 h apart. Culture supernatants were collected at 1, 24, 48, and 72 hpi, and cell lysates were harvested in RLT buffer at 72 hpi.

### 2.5. Human Airway Organoid Culture and Infection

The air–liquid interphase differentiated airway organoid model was built on the airway organoids as previously described [14,17] with a few modifications. Briefly, TrypLE select (Gibco) was used to dissociate the airway organoids into single cells with 10 min incubation at 37 °C. The cells were sheared using a 25G syringe and strained using a 40 μm cell strainer. A total of 200,000 cells were seeded onto a Transwell insert (Corning) pre-coated with rat tail collagen 1 (Corning). The cells were cultured in a mixture of airway organoid growth medium [14,17] and complete base medium (Stemcell Pneumacult-ALI, STEMCELL Technologies Inc., Vancouver, BC, Canada) at a ratio of 1:1 at 37 °C. These were then cultured at the air–liquid interface (ALI) in Maintenance Medium (Stemcell Pneumacult-ALI) after reaching confluency. The Transwell cultures were used for infection after 2 weeks. Cells were first infected with SARS-CoV-2 and then influenza A viruses at MOI 0.1 at the apical side for 1 h at 37 °C with 24 h apart. Cells were washed with PBS and cultured in ALI in the same growth medium.

### 2.6. Viral Titration by TCID_50_ Assay

The viral titers of the influenza A and SARS-CoV-2 viruses were titrated in parallel using 50% tissue culture infectious dose (TCID_50_) assays in the MDCK or Vero E6/TMPRSS2 cells, respectively, except for the replications of SARS-CoV-2 in its co-infection with influenza H5N1, which were assessed with viral ORF1b gene copies measured by quantitative PCR. The 96-well tissue culture plates of the MDCK and Vero E6/TMPRSS2 cells were prepared one day before the TCID_50_ assays. Cells were washed once with PBS and replenished with serum-free MEM supplemented with 2 µg/mL of TPCK-treated trypsin for the MDCK cells, and 2% DMEM for the Vero E6/TMPRSS2 cells. Virus supernatants were serially diluted from 0.5-log to 7-log and added to the cell plates in quadruplicate. The cell plates were incubated in 5% CO_2_ at 37 °C and observed for cytopathic effect (CPE) at 72 hpi. The endpoints of viral dilution leading to the CPE in 50% of the inoculated wells were used to calculate virus titers using the Spearman–Kärber method. The area under the curve (AUC) was calculated by integrating the infectious virus titers at 24–72 hpi.

### 2.7. RNA Extraction and Quantitative PCR

Total RNA was extracted from the harvested human tissue explants using the RNeasy Micro Kit (Qiagen) and from the cell lysates using the MiniBEST Universal RNA Extraction Kit (TaKaRa Bio, Shiga, Japan), according to the manufacturers’ instructions. RNA was reverse-transcribed with oligo-dT and random primers using the PrimeScript RT Reagent Kit (TaKaRa Bio). mRNA expressions of the viral and cytokine genes were quantified with the corresponding gene primers (Supplementary Table S1) using SYBR Premix Ex Taq II (TaKaRa Bio) and measured with a ViiA7 Real-Time PCR System (Applied Biosystems, Waltham, MA, USA). The gene expression levels were quantified with the respective standards and normalized with the β-actin levels.

### 2.8. Cytometric Bead Array (CBA)

The protein levels of the cytokines and chemokines, including human IL-6, IL-8, IP-10, MCP-1, RANTES, and TNF, in the culture supernatants were determined by the multiplexed bead-based BD Cytometric Bead Array Flex Set (BD Biosciences, Heidelberg, Germany) according to the manufacturer’s protocol. In brief, 50 μL of the culture supernatants and concentration standards (ranging from 0 to 2500 pg/mL) were incubated with the capture bead mix for 2 h, followed by incubation with detection antibodies for 1 h. The bead samples were analyzed using a BD LSR Fortessa Analyzer (BD Biosciences). Standard curves for the cytokines and chemokines were built, and the sample concentrations with respect to the fluorescence intensities were calculated using FlowJo version 7.6.1 (BD Life Sciences, Franklin Lakes, NJ, USA).

### 2.9. Immunofluorescence Staining

To indicate the virus-infected cells, Calu-3 and A549/ACE2 cells on glass coverslips were fixed with 4% paraformaldehyde, permeabilized with 0.1% Triton X-100, and blocked with 1% bovine serum albumin. Cells were stained for influenza A viruses with the Nucleoprotein (NP) HB65 antibody and SARS-CoV-2 with Nucleocapsid Antibody (Sino Biological, Wayne, PA, USA), followed by staining with secondary antibodies including the Alexa Fluor 488-conjugated, goat anti-mouse antibody (Invitrogen, Waltham, MA, USA) and Alexa Fluor 594-conjugated, goat anti-rabbit antibody (Invitrogen). The cell nuclei were counterstained with DAPI (BD Biosciences). Afster staining, the sections were mounted with fluorescent mounting medium (Dako, Carpinteria, CA, USA) and imaged using ECLIPSE Ti-S and Ni-E microscopes (Nikon Instruments, Melville, NY, USA). The fluorescence intensities of the SARS-CoV-2 and influenza virus foci were measured with ImageJ software version 1.54p (NIH) and normalized to the intensities of the respective cell nuclei DAPI foci.

### 2.10. Statistics

All experiments were performed at least three times, and the results presented as the mean values ± standard deviation (SD), with the differences considered to be significant at *p* < 0.05. Statistical analysis was conducted using GraphPad Prism version 10 (GraphPad Software, Boston, MA, USA). The viral titers, mRNA expressions, and protein concentrations of the cytokines and chemokines were compared using two-way ANOVA with Tukey’s multiple comparisons test. The area under the curve (AUC) was compared by one-way ANOVA with Tukey’s multiple comparison test.

## 3. Results

### 3.1. SARS-CoV-2 Replication Was Not Affected by Co-Infection with Influenza A Viruses in Human Bronchial Tissues

The Omicron and its sub-lineage variants have been the dominant SARS-CoV-2 strains in Hong Kong as well as worldwide since late 2021. Omicron BA.1 is the parent strain of all subvariants, and this strain has been demonstrated to have superior transmissibility in humans and replication in human bronchial tissues when compared to the previous Delta and wild-type strains [13]. Therefore, we chose Omicron (SCoV2/BA.1) as a representative strain for the investigation of the simultaneous co-infection of SARS-CoV-2 and influenza A viruses including the pandemic H1N1, seasonal H3N2 (4550), seasonal H3N2 (OK/483), and highly pathogenic avian influenza H5N1 in human upper and lower respiratory tract tissues. The viral replications were determined by infectious titers using the TCID_50_ assay, except for the viral load of SARS-CoV-2 upon co-infection with influenza H5N1, where both viruses caused indistinguishable CPE in the Vero E6/TMPRSS2 cells, and thus SARS-CoV-2 replication was determined by ORF1b gene copies using qPCR.

**Figure 1:**
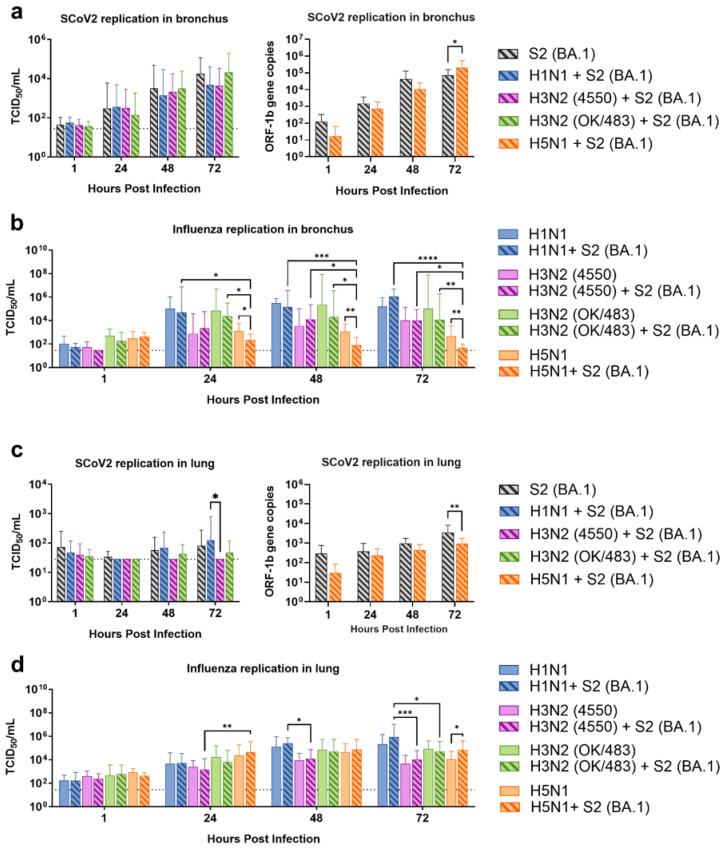
Replication kinetics of influenza A virus and SARS-CoV-2 co-infection in the ex vivo culture of human respiratory tract tissues. Viral replications of SARS-CoV-2 Omicron (BA.1) (**a**,**c**) and influenza A viruses (**b**,**d**) in the human bronchus (**a**,**b**) and lung (**c**,**d**) tissue explants. The viral replications were determined by infectious titers using TCID_50_ assays or SARS-CoV-2 ORF1b gene copies using quantitative PCR. The dotted lines denote the limit of detection in the TCID_50_ assays. Data are presented as the mean values ± SD from 4 individual donors, analyzed with two-way ANOVA followed by Tukey’s test. * *p* < 0.05; ** *p* < 0.01; *** *p* < 0.001; **** *p* < 0.0001.

From the human bronchus explants, we demonstrated that co-infections with the H1N1, H3N2 (4550), H3N2 (OK/483), and H5N1 influenza viruses did not significantly affect the SARS-CoV-2 replication compared with SCoV2/BA.1 single-infection (Figure 1a). On the other hand, co-infection with SCoV2/BA.1 significantly reduced the replication of influenza H5N1 but not the other strains at 24, 48, and 72 hpi (Figure 1b) and in the resultant AUC (Figure S1a) compared with the respective single infections. In the human lung tissue explants, SCoV2/BA.1 was found to have overall lower viral titers than in the bronchus tissues with or without influenza virus co-infections (Figure 1c). There were no significant changes in the replications of influenza H1N1, H3N2, H5N1, or SARS-CoV-2 from the co-infections (Figure S1b), except that the co-infection reduced SCoV2 replication but promoted the influenza H5N1 titers at 72 hpi compared with the respective single infections (Figure 1c,d).

### 3.2. Strain-Specific Effects of Influenza A Virus on the Innate Immune Response of SARS-CoV-2 Infection in Ex Vivo Models

Severe COVID-19 and influenza diseases are associated with the hyper-induction of cytokines. Therefore, we evaluated the innate immune responses, in terms of the mRNA expressions of cytokines and chemokines, in response to the co-infections of the SCoV2 Omicron (BA.1) and influenza viruses using ex vivo models of the human respiratory tract.

**Figure 2:**
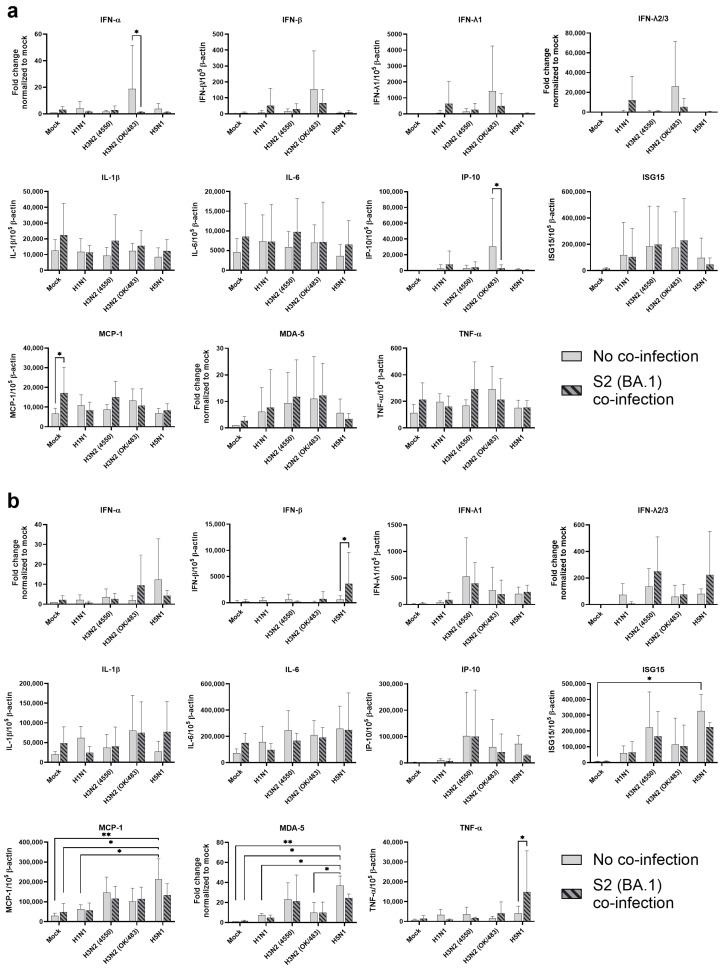
Innate immune responses in human bronchial and lung tissues upon co-infection of SARS-CoV-2 and influenza A viruses. The mRNA expressions of the cytokines and chemokines in human bronchial (**a**) and lung tissues (**b**) infected with SCoV2/BA.1 and/or influenza A viruses, measured with quantitative PCR at 72 h post-infection. Data are presented as the mean values ± SD from 4 individual donors, analyzed with two-way ANOVA followed by Tukey’s test. * *p* < 0.05; ** *p* < 0.01.

In the bronchial tissue lysates, SCoV2/BA.1 infection resulted in a significant induction in the MCP-1 mRNA level compared with the mock-infected samples (Figure 2a). There were no statistical differences in the cytokine expressions between the single infections of different influenza A viruses. However, H3N2 (OK/483) infection showed trends of upregulation in cytokine induction including IFN-α, IFN-β, IFN-λ1, IFN-λ2/3, and IP-10, while co-infection with SCoV2/BA.1 reduced the levels of these cytokines and chemokines. Furthermore, co-infection with H1N1 or H3N2 (OK/483) enhanced the induction of IFNs, IP-10, ISG15, MDA-5 when compared with SCoV2/BA.1 single infection.

For the lung tissue explants, influenza H5N1 virus infection significantly induced the mRNA expression of ISG15, MCP-1, and MDA-5 and trends in the upregulation of IFN-α, IP-10, and IL-6 were also observed when compared with mock infections (Figure 2b). Although SCoV2/BA.1 single infection did not show significant effects on the induction of cytokines, its co-infection with influenza H5N1 resulted in the suppression of all the above cytokines and chemokines. In contrast, co-infection upregulated the induction of IFN-β, IFN-λ2/3, IL-1β, and TNF-α expression. There was an elevated trend in IFN-λ1, IL-6, ISG15, and MCP-1 expression with H3N2 (4550) infection, which were suppressed upon co-infection with SCoV2/BA.1. In general, H3N2 (4550) or H5N1 co-infection enhanced the innate immune responses of SCoV2/BA.1 in lung tissues compared with SCoV2 infection alone.

### 3.3. Differential Susceptibility to SARS-CoV-2 and Influenza A Viruses During Co-Infection in Human Airway and Alveolar Epithelial Cells

**Figure 3:**
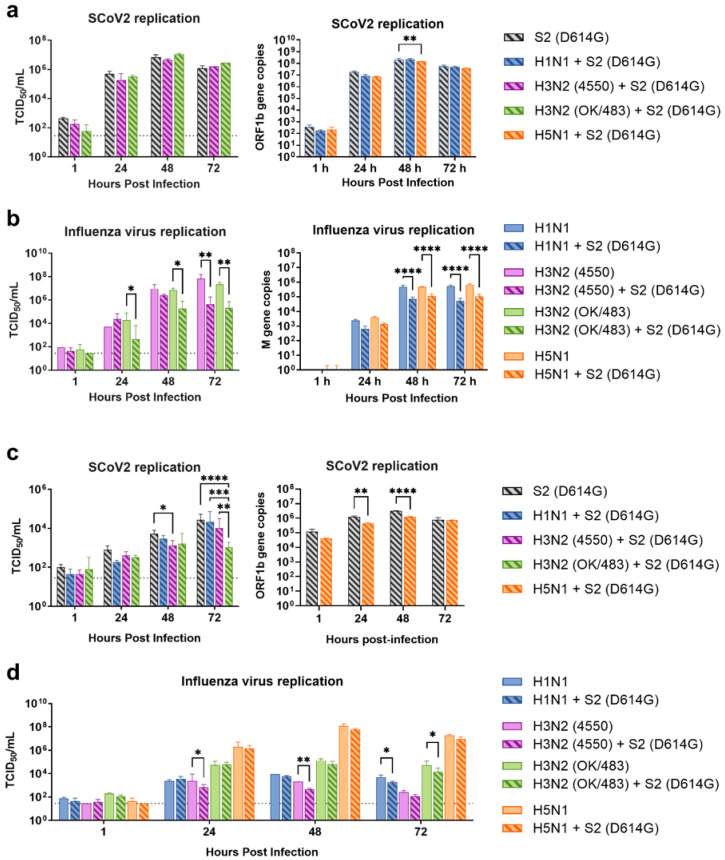
Replication kinetics of influenza A virus and SARS-CoV-2 co-infection in human airway and alveolar epithelial cells. Viral replications of SCoV2/D614G (**a**,**c**) and influenza A viruses (**b**,**d**) in the human airway epithelial cells Calu-3 (**a**,**b**) and alveolar epithelial cells A549/ACE2 (**c**,**d**). The viral replications were determined by infectious titers using TCID_50_ assays or viral gene copies of SARS-CoV-2 (ORF1b) and influenza A (matrix M gene) using quantitative PCR. The dotted lines denote the limit of detection in the TCID_50_ assay. Data are presented as the mean values ± SD (N ≥ 3), analyzed with two-way ANOVA followed by Tukey’s test. * *p* < 0.05; ** *p* < 0.01; *** *p* < 0.001; **** *p* < 0.0001.

**Figure 4:**
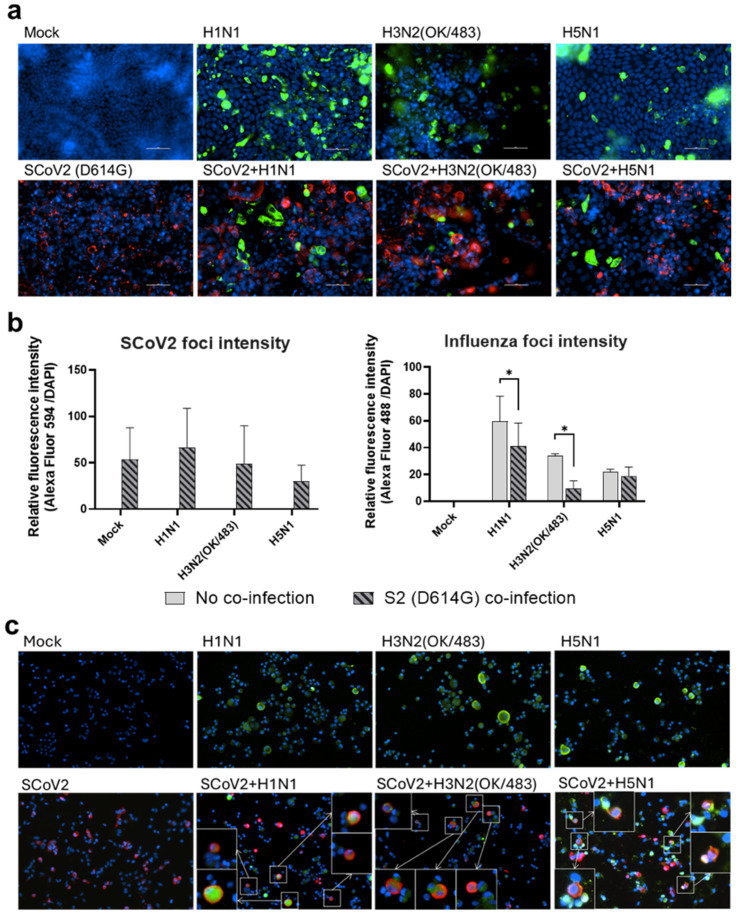
Immunofluorescence staining of SARS-CoV-2 and influenza A viruses. Representative images (200× magnification) of the human airway epithelial cells Calu-3 (**a**) and alveolar epithelial cells A549/ACE2 (**c**) infected with SARS-CoV-2 (SCoV2/D614G) and influenza A viruses, and stained for SCoV2 nucleoprotein (red), influenza A nucleoprotein (green), and cell nuclei (blue). Foci intensities (**b**) of SCoV2 and influenza viruses were measured from the co-infection of Calu-3 cells. Data are presented as the mean values ± SD (N ≥ 3), analyzed with two-way ANOVA followed by Tukey’s test. * *p* < 0.05. Colocalizations of SCoV2 and influenza virus foci (**c**) are indicated by the white squares.

Since the SARS-CoV-2 Omicron (BA.1) variant induced weak immune responses in human lung and bronchial tissues, and due to donor–donor variations, our findings suggest that ex vivo models may not be a suitable model for the assessment of innate immune responses upon virus infection. Therefore, we used the SARS-CoV-2 D614G variant, which has been shown to be a strong cytokine inducer [14], to study its interaction with influenza A viruses during co-infection in the human airway and alveolar epithelial cells. Differentiated airway epithelial Calu-3 cells cultured in the air–liquid interface were used as an in vitro model of the human upper airway. Co-infection of SCoV2/D614G with different influenza A viruses did not have a significant impact on the replication of SARS-CoV-2 in the airway epithelial cells (Figure 3a and Figure S2a). On the other hand, the co-infections significantly reduced the replication of H1N1, H3N2 (4550), H3N2 (OK/483), and H5N1 compared with the corresponding single infections (Figure 3b and Figure S2b). These data suggest that airway epithelial cells are more susceptible to SARS-CoV-2/D614G replication than the influenza A viruses during co-infection. The immunofluorescent staining of the SARS-CoV-2 and influenza A viruses in the airway epithelial cells also illustrated reduced numbers of influenza foci in the co-infections compared with the single infections, confirming the lower replication of influenza A viruses, while there was no prominent reduction in the number of foci stained with the SARS-CoV-2 nucleoprotein (Figure 4a). The quantification of the intensities of SCoV2 and influenza viral antigen staining was consistent with the results of the viral titer measurement (Figure 4b). Furthermore, there was no colocalization of the SARS-CoV-2 and influenza viral protein staining, suggesting that these viruses infect distinct cells during co-infection.

Human alveolar epithelial cells A549/ACE2 were used as an in vitro model to represent the co-infection dynamics in the lower lung. In alveolar epithelial cells, co-infections with the H3N2 (4550), H3N2 (OK/483), and H5N1 influenza viruses significantly reduced the replication of SARS-CoV-2 compared with its single infection (Figure 3c and Figure S2c). Similarly, co-infections also reduced the replication of these influenza A viruses—H1N1 and H3N2 (OK/483) titers at 72 hpi, and H3N2 (4550) viral load (Figure 3d) and the overall AUC levels of H3N2 (4550) and H5N1 (Figure S2d). Immunofluorescent staining of the viral antigen demonstrated colocalization of the SCoV2 and influenza virus infection in the same cells in co-infection with H5N1, H1N1, and H3N2/OK (Figure 4c). Taken together, we demonstrated that SCoV2/D614G and different influenza A viruses compete and suppress each other during co-infections in alveolar epithelial cells.

### 3.4. Elevation of Cytokine Induction by Co-Infection with Influenza A Viruses in Human Airway and Alveolar Epithelial Cells

**Figure 5:**
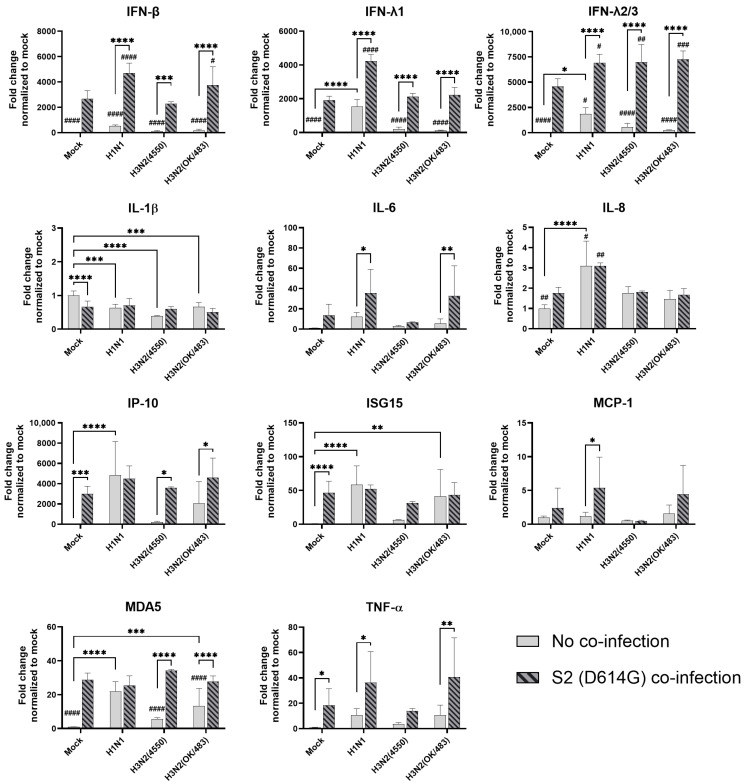
Innate immune responses in human airway epithelial cells co-infected with SARS-CoV-2 and the H1N1 and H3N2 influenza A viruses. The mRNA expressions of the cytokines and chemokines in Calu-3 cells infected with SCoV2/D614G and/or influenza A viruses (H1N1 and H3N2), measured with quantitative PCR at 72 h post-infection. Data are presented as the mean values ± SD (N ≥ 3), analyzed with two-way ANOVA followed by Tukey’s test. Hashtag # denotes the statistical significance in comparison to SCoV2/D614G single infection. # *p* < 0.05; ## *p* < 0.01; ### *p* < 0.001; #### *p* < 0.0001. * *p* < 0.05; ** *p* < 0.01; *** *p* < 0.001; **** *p* < 0.0001.

**Figure 6:**
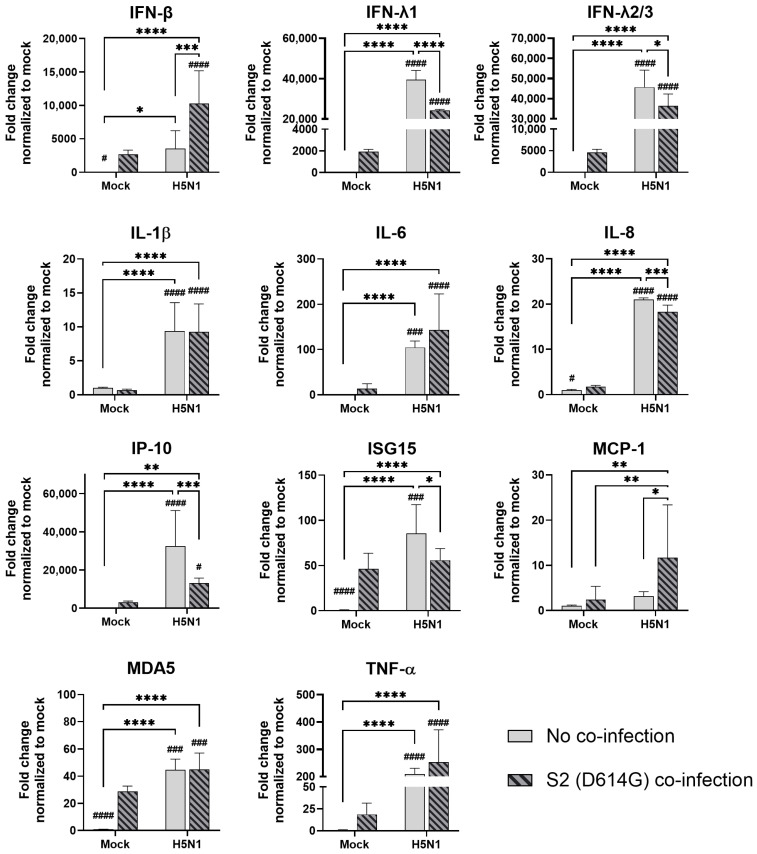
Innate immune responses in human airway epithelial cells co-infected with SARS-CoV-2 and H5N1 influenza A virus. The mRNA expressions of the cytokines and chemokines in Calu-3 cells infected with SCoV2/D614G and/or influenza A viruses (H5N1), measured with quantitative PCR at 72 h post-infection. Data are presented as the mean values ± SD (N ≥ 3), analyzed with two-way ANOVA followed by Tukey’s test. Hashtag # denotes the statistical significance in comparison to SCoV2/D614G single infection. # *p* < 0.05; ### *p* < 0.001; #### *p* < 0.0001. * *p* < 0.05; ** *p* < 0.01; *** *p* < 0.001; **** *p* < 0.0001.

In the airway epithelial Calu-3 cells, multiple cytokine gene expressions were upregulated by the infections of SCoV2/D614G and different influenza A viruses. SCoV2/D614G infection induced the mRNA levels of IFN-β, IFN-λs, IL-8, IP-10, ISG15, MDA5, and TNF-α compared with the mock infection, where the induction of interferons (IFN-β, IFN-λ1, and IFN-λ2/3) and MDA5 were greater than that of the influenza H1N1 and H3N2 infections (Figure 5). H1N1 infection induced IFN-λs, IL-8, IP-10, ISG15, and MDA5 expressions, whilst H3N2 (OK/483) only induced ISG15 and MDA5, and H3N2 (4550) infection showed no immunomodulatory effects. It was noteworthy that the co-infections of SCoV2/D614G with influenza H1N1 resulted in the enhanced gene expression levels of IFN-β, IFN-λ1, IFN-λ2/3, and IL-8 compared with the SCoV2/D614G single infections. Similarly, the co-infection of SCoV2/D614G with influenza H3N2 (OK/483) also resulted in the enhanced gene expression levels of IFN-β, and IFN-λ2/3 compared with the SCoV2/D614G single infections. Avian influenza H5N1 was found to promote all of the tested cytokine and chemokine gene levels except for MCP-1, with an overall higher extent than SCoV2/D614G (Figure 6). The co-infection of SCoV2/D614G with influenza H5N1 also further upregulated the mRNA expression of IFN-β, IL-6, and MCP-1 compared with the respective single infections. The IFN-λs, IL-8, IP-10, and ISG15 expressions were reduced by co-infection when compared with H5N1 single infection. More importantly, all of the tested cytokines and chemokines, except for ISG15, were elevated in co-infection with H5N1 compared with the levels induced by SCoV2/D614G single infection. At the protein level, SCoV2/D614G infection produced higher levels of IP-10 and RANTES but lower levels of MCP-1 and IL-8 compared with the mock infection (Figure S3a). Co-infection of SCoV2 and H1N1 caused an elevation in the IL-6 and IL-8 concentrations compared with the SCoV2 single infections, while the co-infection of SCoV2 and H5N1 caused an elevation in the IL-6, IL-8, IP-10, RANTES, and TNF concentrations than SCoV2 infection.

**Figure 7:**
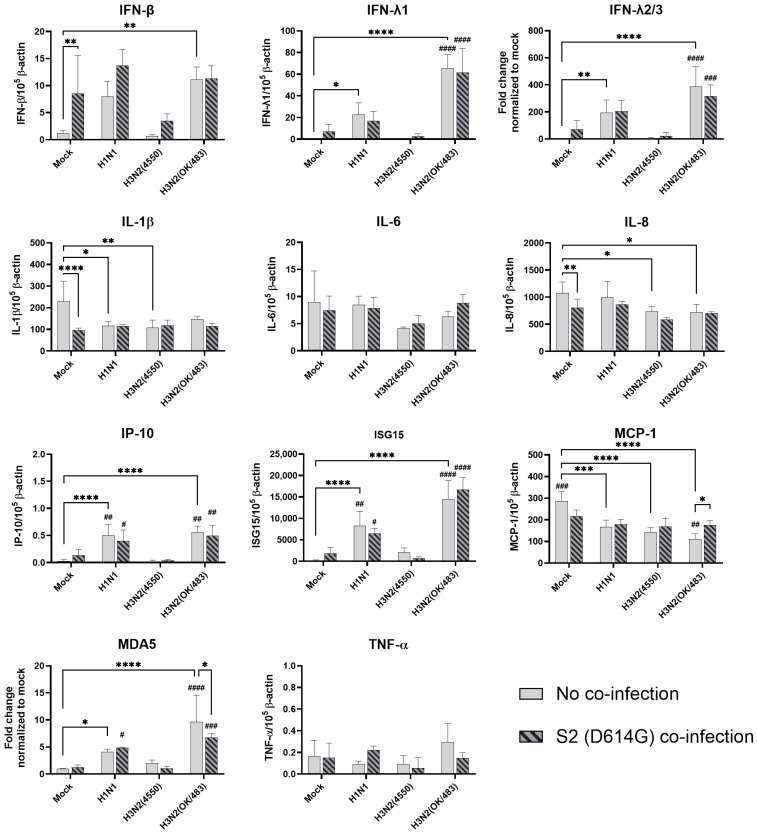
Innate immune responses in human alveolar epithelial cells co-infected with SARS-CoV-2 and the H1N1 and H3N2 influenza A viruses. The mRNA expressions of the cytokines and chemokines in A549/ACE2 cells infected with SCoV2/D614G and/or influenza A viruses (H1N1 and H3N2), measured with quantitative PCR at 72 h post-infection. Data are presented as the mean values ± SD (N ≥ 3), analyzed with two-way ANOVA followed by Tukey’s test. Hashtag # denotes the statistical significance in comparison to SCoV2/D614G single infection. # *p* < 0.05; ## *p* < 0.01; ### *p* < 0.001; #### *p* < 0.0001. * *p* < 0.05; ** *p* < 0.01; *** *p* < 0.001; **** *p* < 0.0001.

**Figure 8:**
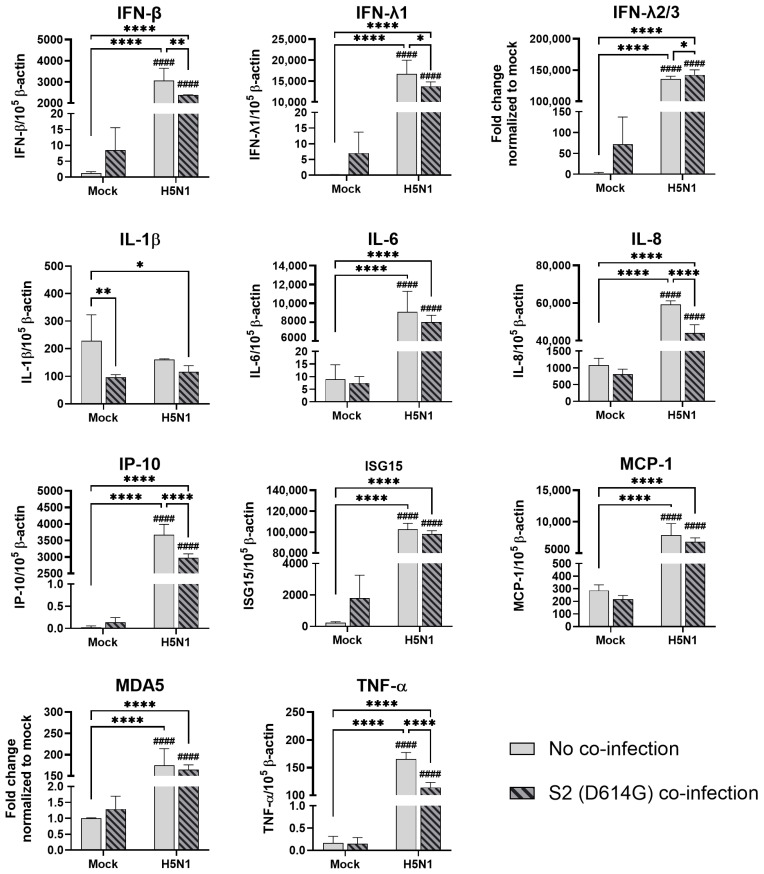
Innate immune responses in human alveolar epithelial cells co-infected with SARS-CoV-2 and H5N1 influenza A virus. The mRNA expressions of cytokines and chemokines in A549/ACE2 cells infected with SCoV2/D614G and/or influenza A virus (H5N1), measured with quantitative PCR at 72 h post-infection. Data are presented as the mean values ± SD (N ≥ 3), analyzed with two-way ANOVA followed by Tukey’s test. Hashtag # denotes the statistical significance in comparison to SCoV2/D614G single infection. #### *p* < 0.0001. * *p* < 0.05; ** *p* < 0.01; **** *p* < 0.0001.

In the alveolar epithelial cells, despite being reported as a strong cytokine inducer, SCoV2/D614G infection showed few inductive effects on the cytokine gene expressions, only IFN-β was upregulated versus mock infection. In contrast, a reduction in IL-1β, IL-8, and MCP-1 expression was observed (Figure 7). On the other hand, influenza H1N1 and H3N2 (OK/483) infections significantly elevated the IFN-λ1, IFN-λ2/3, IP-10, ISG15, and MDA5 mRNA levels compared with the mock infection. H5N1 infection robustly induced the mRNA levels of IFN-β, IFN-λs, IL-6, IL-8, IP-10, ISG15, MCP-1, MDA5, and TNF-α but not IL-1β (Figure 8). Co-infection of SCoV2 with H3N2 (OK/483) led to enhanced levels of IFNs, IP-10, ISG15, and MDA5 than that by SCoV2 infection while H1N1 co-infection enhanced IP-10, ISG15, and MDA5 compared with SCoV2 single infection (Figure 7). Furthermore, all of the tested cytokines and chemokines were elevated during the co-infection of SCoV2/D614G with influenza H5N1 compared with SCoV2 single infection (Figure 8).

The cytometric bead array analysis confirmed the enhancement in the cytokine and chemokine concentrations by the SCoV2/D614G and influenza H5N1 infections in the alveolar epithelial cells (Figure S3b). Only influenza H5N1 infection significantly induced the IL-6, IL-8, IP-10, RANTES, and TNF concentrations.

### 3.5. Angiotensin-Converting Enzyme 2 Levels in Response to SARS-CoV-2 and Influenza A Virus Infection in Human Airway and Alveolar Epithelial Cells

Given that angiotensin-converting enzyme 2 (ACE2) plays an important role in SARS-CoV-2 infection, taking part in virus entry and host homeostasis [18], we also investigated the ACE2 expression changes under SARS-CoV-2 and influenza A virus infections. In the airway epithelial cells, SCoV2/D614G suppressed the expression of the ACE2-long form receptor of SARS-CoV-2 in single infections and co-infection with H1N1 or H3N2 (Figure S4a). We also found that SCoV2/D614G, influenza H1N1, and H5N1 infections significantly induced the short soluble form of ACE2 compared with the mock infection (Figure S4a). SCoV2/D614G upregulated the expression of the soluble form of ACE2 to a similar level as that of H5N1 and at significantly higher levels than the other influenza A viruses.

However, only influenza H5N1 infection and its co-infection upregulated both the long and short isoforms of ACE2 in the alveolar epithelial cells (Figure S4b), while H1N1 and SCoV2/D614G co-infection had a trend of upregulation of the short isoform of ACE2. Co-infection of SCoV2/D614G with H5N1 enhanced the expression of the ACE2 long and short isoform compared with SCoV2 single infection.

### 3.6. Sequential Infection of SCoV2 and Influenza A in Airway and Alveolar Epithelial Cells

**Figure 9:**
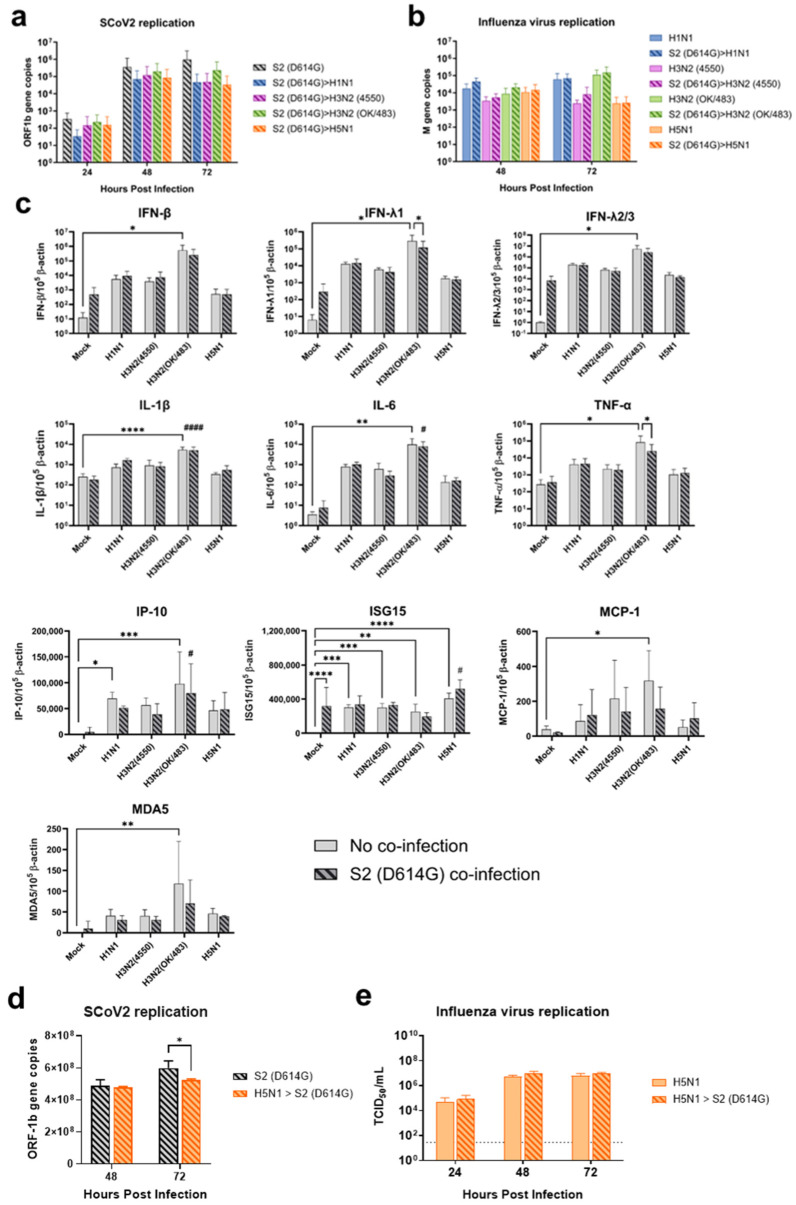
Sequential infection of influenza A virus and SARS-CoV-2 in the human airway organoid and alveolar epithelial cells. Viral replications of the SCoV2/D614G (**a**) and influenza A viruses (**b**) and the mRNA expressions of cytokines and chemokines (**c**) in human airway organoid culture sequentially infected with SCoV2/D614G followed by influenza A viruses. Viral replications in A549/ACE2 cells (**d**,**e**) sequentially infected with influenza H5N1 followed by SCoV2/D614G. The dotted line denotes the limit of detection in the TCID_50_ assays. Data are presented as the mean values ± SD (N ≥ 3), analyzed with two-way ANOVA followed by Tukey’s test. Hashtag # denotes the statistical significance in comparison to SCoV2/D614G single infection. # *p* < 0.05; #### *p* < 0.0001. * *p* < 0.05; ** *p* < 0.01; ***p < 0.001; **** *p* < 0.0001.

Sequential infection with SCoV2 and then influenza virus at 24 hpi in the airway epithelial cells showed a trend of reduction in SCoV2 replication during co-infection, particularly co-infection with H5N1, without statistical significance, while there was no change in the replication of the influenza viruses (Figure 9a,b and Figure S2e). H3N2 (OK/483) infection induced the highest levels of cytokine and chemokine expression and the ACE2-long form among all of the tested viruses (Figure 9c and Figure S4c). However, we found that H5N1 co-infection induced the highest level of ISG15, which may be the reason for the observed slight reduction in SCoV2 replication.

Since the ACE2-long form was upregulated in the A549/ACE2 cells upon H5N1 infection (Figure S4b), we wondered whether pre-infection with H5N1 and subsequent infection with SCoV2 would enhance the replication of the latter. After 24 hpi with the H5N1 virus, SCoV2 viruses were added to the A549/ACE2 cells. The replication of SCoV2 did not increase; in contrast, there was a slight reduction with co-infection of the H5N1 virus at 72 hpi (Figure 9d and Figure S2g). There was no change in the replication of H5N1 (Figure 9e and Figure S2h).

## 4. Discussion

After the COVID-19 pandemic, many countries moved away from strict “zero-infection” policies and loosened their prevention measures in order to begin normalizing global communication and economic activities. Despite this shift, the Omicron variants remained the dominant SARS-CoV-2 strains continuing to circulate worldwide. Concurrently, the normal seasonal patterns of influenza A virus activity reemerged in many regions after the suppression observed earlier in the COVID-19 pandemic. While previous research mainly focused on co-infections with early strains of SARS-CoV-2 and showed that co-infection with COVID-19 variants and influenza could lead to more severe illness and higher mortality rates, more recent studies using Omicron variants have shown diverse findings in susceptibility and severity. A study demonstrated that viral co-infection in COVID-19 patients was associated with elevated ICU admission and mortality where influenza A H1N1 had a direct relationship with mortality in these patients [19]. However, another study with 9498 patients reported that there was no effect on the overall mortality as well as a lower risk of critical outcomes in co-infection patients [20]. These findings suggest that further studies on different influenza virus strains and different orders of infection sequence should be performed. Furthermore, this discrepancy may be attributed to the infection models and the differences in the infection experimental design and schedules. One report showed that Omicron infection reduced influenza replication via the induction of IFN responses in a sequential infection setting in which bronchial cells cultured in an air–liquid interface were infected with Omicron for 48 h before exposure to an influenza A virus [11]. This study only represented one of the co-infection scenarios, and the antiviral effects mounted by pre-infection with Omicron probably masked the replication of the influenza A virus, hence leading to missing information when the cells were susceptible to both SARS-CoV-2 and influenza A virus infection. Another group using Matrigel-based alveolar organoids composed predominantly of alveolar type II cells showed that Delta variant infection enhanced the influenza viral load [12]. However, Matrigel is only penetrable to the SARS-CoV-2 virus but not the influenza A virus, which may cause bias on the replicating conditions to the two different virus strains, and as the organoids are mainly composed of alveolar type II cells, this model may not reflect the physiological situations in the human lower lung upon co-infection.

To address this knowledge gap, we used ex vivo models of the human respiratory tract to investigate the potential impacts of Omicron and influenza co-infection, given the continued circulation of both viruses in the post-pandemic environment. In order to investigate the interaction between these viruses in a comprehensive context, we used various influenza A viruses including the highly transmissible pandemic H1N1, seasonal H3N2, and highly pathogenic avian influenza H5N1 viruses. Although H5N1 infection is relatively rare, this virus leads to a high mortality rate and severe lower respiratory tract infection. In human bronchial tissues, we found that the influenza A viruses did not affect the replication of the Omicron variant of SARS-CoV-2. However, there was a trend of reduced replication of the influenza A virus in the co-infection setting. While the Omicron variant replicated at lower levels compared with the influenza A virus in a single infection setting in lung tissues, the co-infection did not affect the replication of each other, except for the titer of H5N1 virus, which was reduced at 72 hpi. This may be due to different target cells in the lung—SARS-CoV-2 mainly infects ACE2 positive type-II cells, while the influenza A virus predominantly infects type-I epithelial cells. In contrast, in the alveolar epithelial cells, co-infection of the two viruses reduced the replication of each other, except for the replication of H1N1pdm. These findings may be due to the viruses competing for the same target cells in the cell line model while having a differential preference of target cells in the lung tissue explants.

Several studies have shown similar findings where there was no change or a reduction in the replication of SARS-CoV-2 during co-infection with the influenza A virus [10,21,22,23]. Co-infection or pre-infection with the H3N2 virus had no effect on the replication of SARS-CoV-2 in Syrian hamsters [21], while pre-infection or co-infection with pandemic H1N1 reduced the replication of SARS-CoV-2 in A549-ACE2 cells [10] in ferrets [22] and in golden Syrian hamsters [23]. In contrast, some studies have reported that co-infection or pre-infection with the influenza virus enhanced the replication of the influenza virus [24] or SARS-CoV-2 [9]. However, these studies used the mouse-adapted H1N1 pandemic, which has different, usually elevated, severity from human infection, in K18-hACE2 transgenic mice [24] or golden Syrian hamsters [23], while the other report used WSN H1N1 in A549 cells, showing a lowered viral titer of SASRS-CoV-2 in the culture supernatant [9].

Previous studies have shown that Omicron replicated less efficiently in the lungs compared with the wild-type strain [16], and most of the SARS-CoV-2 infections did not upregulate the host innate responses, leading to less severe pneumonia [25,26]. However, SARS-CoV-2 has been continuously circulating and evolving in human populations. In recent years, two descendant variants of the BA.2 sublineage—BA.2.86/EG.5.1 and JN.1—have emerged and surpassed the XBB variants to become the predominant variants. Although recent Omicron variants have a low ability to induce a high level of cytokines, there is a chance that a previous lineage with high cytokine induction will return to human populations. In addition to its immune evasion ability, it is important to investigate the interactions of SARS-CoV-2 with a high cytokine phenotype together with different strains of influenza A viruses to better prepare for future outbreaks. Therefore, we investigated the co-infection of a variant D614G that elicits strong immune responses [14] with various influenza A viruses to better understand the interactions between a high cytokine SARS-CoV-2 variant and these influenza A viruses in a co-infection setting.

In human airway epithelial cells, we found that SARS-CoV-2 induced higher levels of cytokines and chemokines than H3N2 (4550), H3N2 (OK/483), and H1N1pdm but lower levels than that by H5N1. Although the specific cytokines and chemokines upregulated by the co-infection were different, in general, the H3N2, H1N1pdm, and H5N1 viruses enhanced the induction of cytokines and chemokines in SARS-CoV-2 infection. In contrast, SARS-CoV-2 did not induce cytokines or chemokines, except for IFN-β in the human alveolar epithelial cells. H1N1, H3N2 (OK/483), or H5N1 co-infection with SARS-CoV-2 elevated the expression of IFN-β, IFN-λ1, IFN-λ2/3, and ISG15, which has been shown to be associated with pathology in severe COVID-19 [27,28,29] and pro-inflammatory cytokines and chemokines upon H5N1 co-infection. These findings suggest that SARS-CoV-2 co-infection with pandemic, seasonal, and avian influenza A viruses probably leads to enhanced disease severity, especially lung disease associated with immunopathology. This elevation may not directly relate to viral replication, since there was no prominent change in SARS-CoV-2 replication while a reduction in viral replication was observed in all of the influenza A viruses tested upon co-infection. Our findings are consistent with available reports on the co-infection of influenza (H1N1 and H3N2) and SARS-CoV-2, regardless of whether they were in vitro or in vivo models with different species of animals, that there are enhanced severities with the pre-infection or co-infection of the two viruses [9,21,22,23,24,30,31]. However, one study reported that co-infection with H1N1pdm did not upregulate the pro-inflammatory response of SARS-CoV-2 but rather induced similar levels of chemokines as SARS-CoV-2 single infection in golden hamsters [10].

There are some explanations on the reduced replication of the influenza A virus and enhanced severity in infected animals. The interference with the replication of influenza by co-infection with SARS-CoV-2 can be attributed to the upregulation of MX1 gene expression by SARS-CoV-2 infection, where MX1 inhibited the replication of the influenza A virus while the upregulation of MX1 by influenza infection did not affect the replication of SARS-CoV-2 [21]. Another report demonstrated that co-infection caused rapid infiltrations of monocytes, B cells, CD4^+^ T cells, and CD8^+^ T cells in the bronchoalveolar lavage fluid in infected mice and led to severe lymphopenia in peripheral blood, which is strongly associated with the impaired production of IgG, and neutralizing antibodies against SARS-CoV-2 and the influenza virus, which increased disease severity [31]. Lower serum SARS-CoV-2 neutralizing antibodies and longer SARS-CoV-2 shedding were also reported by another group using golden Syrian hamsters [23].

The ACE2 protein is a critical component in the pathogenesis of COVID-19, which is caused by the SARS-CoV-2 virus. ACE2 serves as the primary receptor to which the SARS-CoV-2 virus binds to gain entry and infect human cells. Some studies have suggested that smoking can upregulate ACE2 expression to increase the severity of patients [32]. Older age and certain underlying health conditions, such as diabetes, cardiovascular disease, and obesity, have been identified as factors that can influence the ACE2 expression levels, which may contribute to increased susceptibility and more severe COVID-19 outcomes in these populations. However, recent research has identified two main isoforms of the ACE2 protein—long and short forms [33]. The long form ACE2 is anchored on the cell membrane with its transmembrane domain, which is the cell entry receptor of SARS-CoV-2, while the short form ACE2 lacking the membrane-binding motifs is characterized as a soluble circulating protein that freely moves and is distributed throughout the extracellular fluid compartments of the body. Although we found that H5N1 induced higher levels of the ACE2 receptor (long form of ACE2) than SARS-CoV-2 infection alone, we did not observe an enhancement in the SARS-CoV-2 viral titers upon co-infection with H5N1 in the alveolar epithelial cells (Figure 3c and Figure S2c). Similar observations were seen in H1N1 and its co-infection in the alveolar epithelial cells. Similarly, a slight reduction in SCoV2 replication was observed when cells were exposed to H5N1 infection and subsequently infected with SCoV2 in the alveolar epithelial cells. These findings suggest that the upregulation of the ACE2-long form (SCoV2 receptor) by H5N1 infection did not enhance the replication of SCoV2; on the other hand, due to the upregulation of ISG15 and IFNs, the replication of SCoV2 was reduced. However, a reduction in virus replication may not represent a reduction in disease severity, since there was an elevation in the number of cytokines and chemokines, which can contribute to immunopathology in the COVID-19 disease. These results suggest that upregulation of the entry receptor is not sufficient to enhance the virus replication of SARS-CoV-2.

While the short soluble form of ACE2 may have some protective effects, such as the potential to neutralize SARS-CoV-2 particles, it modulates the immune response by stimulating the production of cytokines and other immune mediators [34]. This is a critical consideration in co-infection situations, as the upregulation of short-form ACE2 by influenza in H1N1 and H5N1 co-infection may cause an overwhelming production of cytokines and other immune-related factors like cytokine storm. Therefore, an overproduction of cytokines and other immune mediators induced by the short-form ACE2 could paradoxically worsen the condition of patients with influenza A virus co-infection.

Various respiratory models were employed in this study. Human bronchial explants are the most physiologically relevant model to represent the human airway, however, limitations include the tissue availability, which also affects the scale of the experimental settings, and a lack of information on the tropism of the apical or basolateral release of virus particles. In contrast, ALI cultures provide a better resolution of virus tropism based on the different compartments of a Transwell. Another limitation of using lung tissues is that viruses spread by diffusion or through other mobile cells such as white blood cells in the human body. In our ex vivo model, viruses are relatively easier to spread from cell to cell, and viruses released from different compartments of the lung tissues may be mixed during handling procedures, which may have an enhancement effect on virus replication. However, this problem can be minimized when all the tissues are handled with the same procedures to achieve a fair comparison.

Although the COVID-19 pandemic has passed and new variants display milder symptoms compared with the wild type and prior variants, the SARS-CoV-2 virus continues to evolve, and newly emerging variants are acquiring immune evasion properties. It is inevitable that SARS-CoV-2 and the influenza A virus will co-exist and co-circulate in human populations. We report here that co-infection with the influenza A virus had minimal effects on the replication and transmission of Omicron/BA.1 in respiratory explants, while influenza replication was reduced in the bronchi and airway epithelial cells. These findings suggest that SARS-COV-2 has a replication advantage over the influenza A virus in the upper airway. Furthermore, there was no significant interference in their replication in the lung tissues. Despite a mutual reduction in viral load being found during co-infection in the alveolar epithelial cells, influenza A virus infection elevated the cytokine responses of SARS-CoV-2 in both the airway and alveolar tissues and cells, especially for infection with the H5N1 and seasonal H3N2 (OK/483) viruses in the alveolar epithelial cells, which may lead to more severe damage to the host than SARS-CoV-2 single infection. Moreover, upregulation of the ACE2 receptor did not promote elevated SARS-CoV-2 replication in the alveolar epithelial cells, while the induction of the soluble form of ACE2 could have further enhanced the cytokine responses induced by H5N1. Taken together, our study provides insights that SARS-CoV-2 has a replication advantage over the influenza A virus in the upper airway and a more severe respiratory disease in the lower lung when there is a co-infection of SARS-CoV-2 and the influenza A virus. Therefore, these findings support the necessity of seasonal influenza vaccination and in maintaining good hygiene practices for reducing the disease severity caused by SARS-CoV-2 and influenza co-infection. Moreover, therapeutic options should be rigorously explored and developed that not only focus on antivirals, but also on immuno-modulatory regimens.

## Data Availability

The original contributions presented in the study are included in the article, further inquiries can be directed to the corresponding author.

## Supplementary Materials

The following supporting information can be downloaded at: https://www.mdpi.com/article/10.3390/microorganisms13050988/s1, Table S1: List of primers used for quantitative PCR; Figure S1: Replication kinetics of influenza A virus and SARS-CoV-2 co-infection in ex vivo culture of human respiratory tract tissues; Figure S2: Replication kinetics of influenza A virus and SARS-CoV-2 co-infection in human airway and alveolar epithelial cells; Figure S3: Cytokine production in human airway and alveolar epithelial cells upon the co-infection of SARS-CoV-2 and influenza A virus; Figure S4: ACE2 expression in human airway and alveolar epithelial cells infected with SARS-CoV-2 and influenza A virus.
