# Supplementary material for: Dynamic Interaction Between SARS-CoV-2 and Influenza A Virus Infection in Human Respiratory Tissues and Cells
Source: Microorganisms. 2025 Apr 25;13(5):988. doi: 10.3390/microorganisms13050988 (PMC12114508; doi:10.3390/microorganisms13050988)
Supplement: Supplementary file 1 [file microorganisms-13-00988-s001.zip › microorganisms-3504217-supplementary.pdf]

Dynamic Interaction Between SARS-CoV-2 and Influenza A Virus Infection in Human Respiratory Tissues and Cells

John C. W. Ho [1,2], Kachun Ng [1], Rachel H. H. Ching [1,2], Malik Peiris [1,2], John M. Nicholls [3], Michael C. W. Chan [1,2] and Kenrie P. Y. Hui [1,2*]

[1]School of Public Health, Li Ka Shing Faculty of Medicine, The University of Hong Kong, Pokfulam, Hong Kong SAR, China;

[2]Centre for Immunology and Infection (C2i), Hong Kong Science Park, Shatin, Hong Kong SAR, China;

[3]Department of Pathology, School of Biomedical Sciences, Li Ka Shing Faculty of Medicine, The University of Hong Kong, Pokfulam, Hong Kong SAR, China.

Supplementary Table S1. List of primers used for quantitative PCR

Gene	Primer sequence (5'-3')
β-actin	F: TGGATCAGCAAGCAGGAGTATG
	R: GCATTTGCGGTGGACGAT
SARS-CoV-2 ORF1b	F: TGGGGYTTTACRGGTAACCT
	R: AACRCGCTTAACAAAGCACTC
Influenza M-gene	F: CTTCTAACCGAGGTCGAAACG
	R: GGCATTTTGGACAAAKCGTCTA
ACE2-long isoform	F: CAAGAGCAAACGGTTGAACAC
	R: CCAGAGCCTCTCATTGTAGTCT
ACE2-short isoform	F: GTGAGAGCCTTAGGTTGGATTC
	R: TAAGGATCCTCCCTCCTTTGT
IFN-α	F: AGAAGGCTCCAGCCATCTCTGT
	R: TGCTGGTAGAGTTCGGTGCAGA
IFN-β	F: CAACTTGCTTGGATTCCTACAAAG
	R: TGCCACAGGAGCTTCTGACA
IFN-λ1	F: GCCCCCAAAAAGGAGTCCG
	R: AGGTTCCCATCGGCCACATA
IFN-λ2/3	F: TTTAAGAGGGCCAAAGATGC
	R: TGGGCTGAGGCTGGATACAG
IL-1β	F: CACGATGCACCTGTACGATCA
	R: GTTGCTCCATATCCTGTCCCT
IL-6	F: GCATGGGCACCTCAGATTGT
	R: TGCCCAGTGGACAGGTTTCT
IL-8	F: CACCGGAAGGAACCATCTCA
	R: AGAGCCACGGCCAGCTT
IP-10	F: ATTATTCCTGCAAGCCAATTTTG
	R: TCACCCTTCTTTTTCATTGTAGCA
ISG15	F: CAAATGCGACGAACCTCTGA
	R: CCGCTCACTTGCTGCTTCA
MCP-1	F: CAAGCAGAAGTGGGTTCAGGAT
	R: TCTTCGGAGTTTGGGTTTGC
MDA-5	F: TCACAAGTTGATGGTCCTCAAGT
	R: CCTTCTCCAGATTTGGCTGAAC
TNF-α	F: GCAGGTCTACTTTGGGATCATTG
	R: GCGTTTGGGAAGGTTGGA

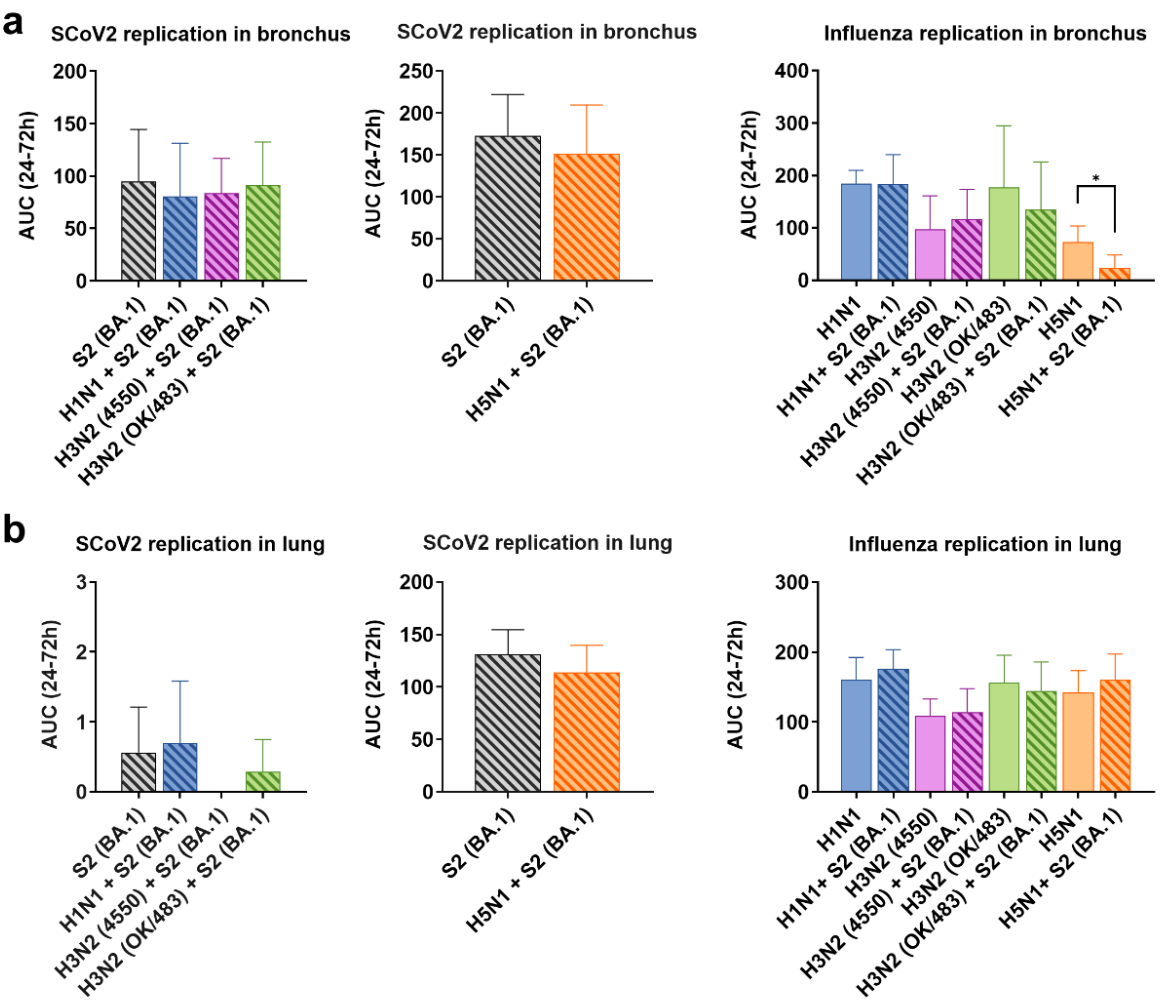

Supplementary Figure S1. Replication kinetics of influenza A virus and SARS-CoV-2 co-infection in ex vivo culture of human respiratory tract tissues. The area-under-curve (AUC) depicted from the viral replication of SARS-CoV-2 Omicron (SCoV2/BA.1) and influenza A viruses in human bronchus **(a)** and lung **(b)** tissue explants. The viral replications were determined by infectious titres using $TCID_{50}$ assays or SARS-CoV-2 ORF1b gene copies using quantitative PCR. Data are presented as the mean values ± SD from 4 individual donors, analyzed with one-way ANOVA followed by Tukey's test. $^{*}p < 0.05$.

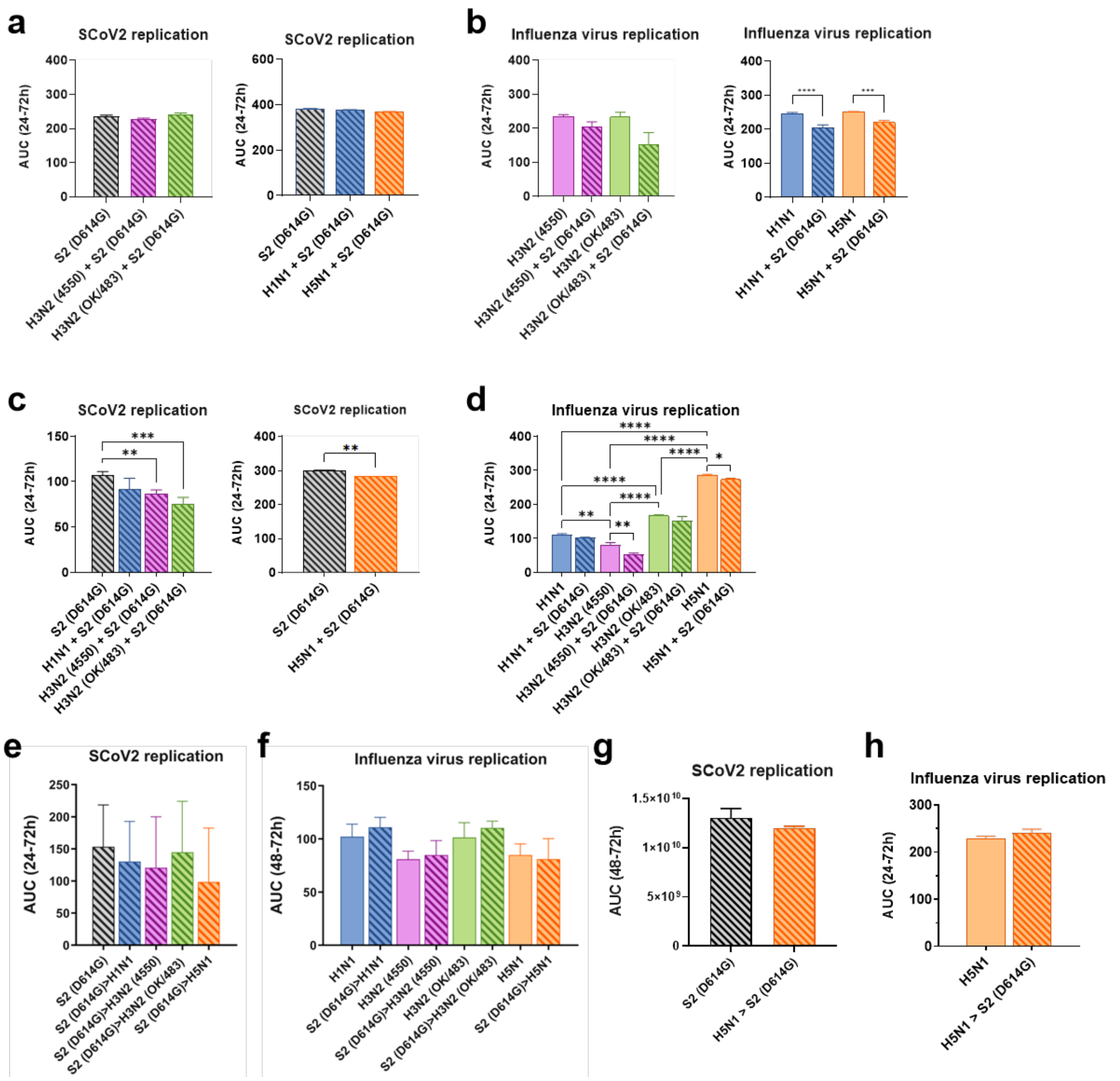

Supplementary Figure S2. Replication kinetics of influenza A virus and SARS-CoV-2 co-infection in human airway and alveolar epithelial cells. The area-under-curve (AUC) depicted from the viral replications of SCoV2/D614G and influenza A viruses in human airway epithelial cells Calu-3 **(a and b)** and alveolar epithelial cells A549/ACE2 **(c and d)** from simultaneous co-infection; in human airway organoid culture **(e and f)** sequentially infected with SCoV2/D614G followed by influenza A viruses, and in A549/ACE2 cells **(g and h)** sequentially infected with influenza H5N1 followed by SCoV2/D614G. Data are presented as the mean values ± SD (N ≥3), analyzed with one-way ANOVA followed by Tukey's test. $^{*}p < 0.05$; $^{**}p < 0.01$; $^{***}p < 0.001$; $^{****}p < 0.0001$.

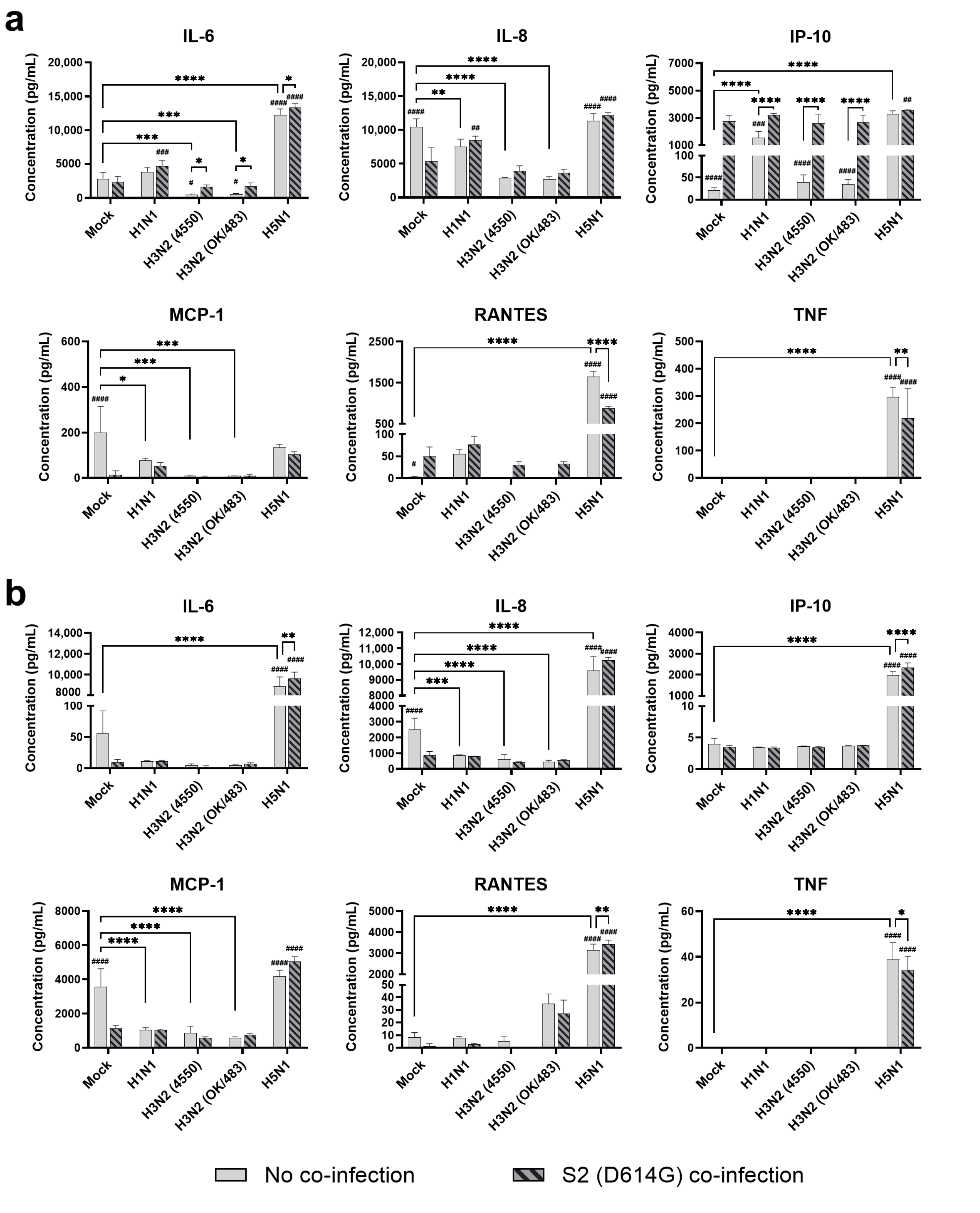

Supplementary Figure S3. Cytokine productions in human airway and alveolar epithelial cells upon co-infection of SARS-CoV-2 and influenza A virus. The protein levels of cytokines and chemokines in cell culture of Calu-3 **(a)** and A549/ACE2 **(b)** infected with SCoV2/D614G and/or

influenza A virus strains, measured with Cytometric Bead Array at 72 h post-infection. Data are presented as the mean values ± SD ($N \geq 3$), analyzed with two-way ANOVA followed by Tukey's test. Hashtags # denote the statistical significance in comparison to SCoV2/D614G single infection. # $p < 0.05$; ## $p < 0.01$; ### $p < 0.001$; #### $p < 0.0001$. * $p < 0.05$; ** $p < 0.01$; *** $p < 0.001$; **** $p < 0.0001$.

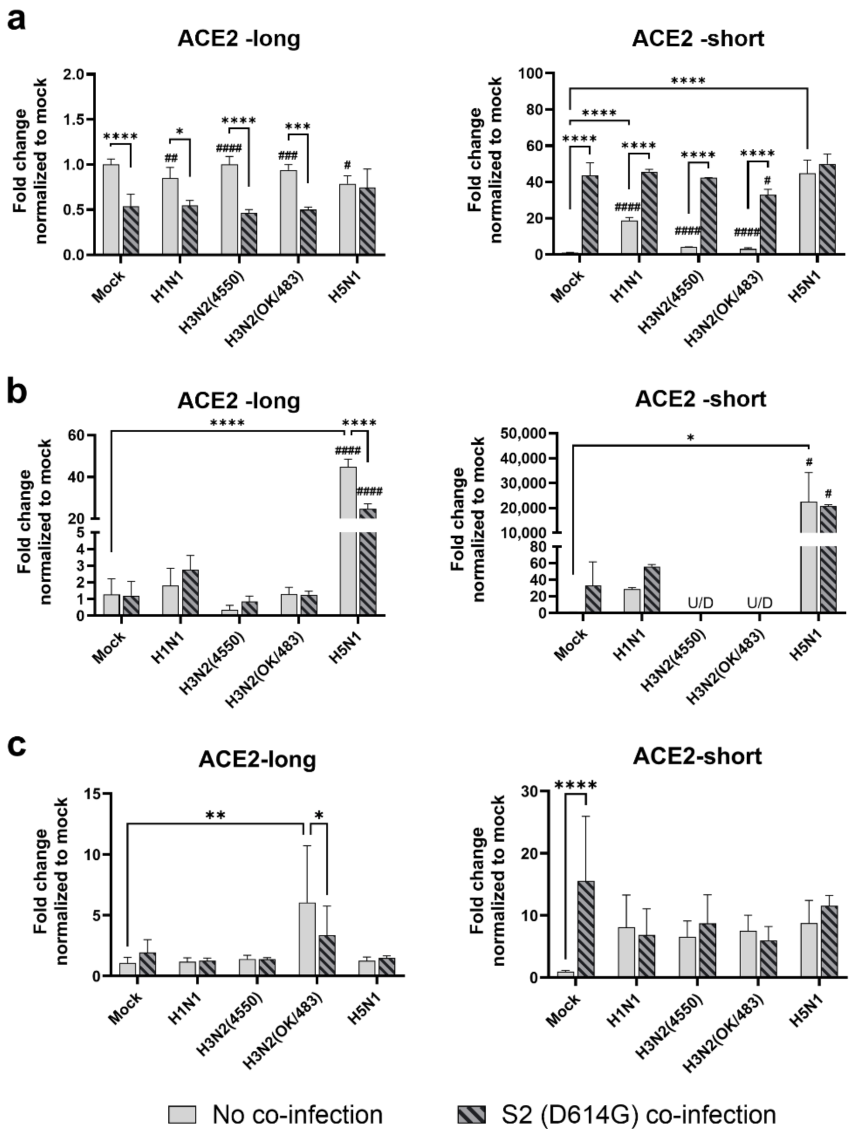

Supplementary Figure S4. ACE2 expression in human airway and alveolar epithelial cells infected with SARS-CoV-2 and influenza A virus. The mRNA expressions of long and short isoforms of ACE2 gene in Calu-3 cells **(a)**, A549/ACE2 cells **(b)**, and human airway organoid culture **(c)** infected with SCoV2/D614G and/or influenza A virus strains, measured with quantitative PCR at 72 h post-infection. Simultaneous co-infection **(a and b)** of SCoV2/D614G and influenza viruses, and sequential infection **(c)** with SCoV2/D614G followed by influenza A viruses at 24 hpi. Data are presented as the mean values ± SD ($N \geq 3$), analyzed with two-way ANOVA followed by Tukey's test. Hashtags # denote the statistical significance in comparison to SCoV2/D614G single infection. # *$p < 0.05$; ##$p < 0.01$; ###$p < 0.001$; ####$p < 0.0001$.* $^{*}p < 0.05$; $^{**}p < 0.01$; $^{***}p < 0.001$; $^{****}p < 0.0001$.
